# Myositis autoantibodies in a racially diverse population of children with idiopathic inflammatory myopathies

**DOI:** 10.1186/s12969-021-00574-6

**Published:** 2021-06-12

**Authors:** Megan Mariko Perron, Natalia Vasquez-Canizares, Gabriel Tarshish, Dawn M. Wahezi

**Affiliations:** 1 Children’s Hospital at Montefiore, 3415 Bainbridge Ave, Bronx, NY 10467 USA; 2 Children’s Hospital Colorado, 13123 East 16th Avenue, Aurora, CO 80045 USA

**Keywords:** Juvenile dermatomyositis, Juvenile polymyositis, Myositis specific autoantibodies

## Abstract

**Background:**

Juvenile idiopathic inflammatory myopathies (JIIMs) is a group of autoimmune disorders, including juvenile dermatomyositis (JDM), juvenile polymyositis (JPM) and overlap myositis, that are characterized by proximal muscle weakness, elevated levels of serum muscle enzymes, and pathognomonic skin findings. While the exact etiology of JIIMs is unclear, the presence of myositis specific autoantibodies (MSAs) have been associated with certain clinical phenotypes, organ involvement and disease prognosis. To date, there have been few studies of the associations between MSA presence and patient ethnicity. It is important to understand the extent to which ethnicity impacts disease manifestations, organ involvement and clinical outcomes. The goal of our study is to determine MSA and myositis associated autoantibody (MAA) presence, clinical phenotype, and disease course in a racially diverse population of pediatric patients with JIIMs.

**Methods:**

Patients age 2–21 years with a prior diagnosis of JDM, JPM or overlap myositis, who had been tested for MSA/MAA, were eligible for study inclusion. Clinical and laboratory data were collected retrospectively via manual chart review in this single-center study. Descriptive statistics were performed to summarize each variable. Given the small sample size, non-parametric testing was performed using Fischer’s exact test, Wilcoxon rank sum test and Kruskal-Wallis test.

**Results:**

Thirty one patients were included in the analysis. Race and ethnicity were self-reported as Hispanic (48.4%), white (25.8%), and Black (25.8%). The most prevalent MSAs were anti-MDA5 (25.8%), anti-p155/140 (22.6%) and anti-MJ (19.4%). Presence of autoantibodies (*p* = 0.04) and pulmonary disease (*p* = 0.03) were significantly higher in patients of Black or Hispanic descent compared with white descent. Anti-MDA5 antibodies, cutaneous ulceration, cardiopulmonary involvement, hospitalizations and one death were only reported in patients with Black or Hispanic descent. Patients with anti-MDA5 antibodies were more likely to be male (p = 0.04) and to have cutaneous ulceration (*p* = 0.02).

**Conclusions:**

This study describes the prevalence of MSA/MAA in a racially diverse group of patients with JIIM and further delineates clinical phenotype and disease complications in these groups. We found a relatively high proportion of children with anti-MDA5 antibodies and described potentially worse clinical courses in children of Black or Hispanic descent. Further investigation is warranted to examine these findings.

## Background

Juvenile idiopathic inflammatory myopathies (JIIMs) are a group of systemic autoimmune diseases characterized by muscle weakness, inflammation and characteristic skin rashes. The most prevalent JIIM is juvenile dermatomyositis (JDM), followed by overlap myositis and juvenile polymyositis (JPM) [1]. JDM is a rare and often chronic disease, with an estimated incidence of 3.2 cases per million per year in the United States and an average age of onset at 7 years of age [2, 3]. While the exact etiology of JIIM remains unknown, autoantibody production may play a role in the underlying pathogenesis and have been linked to distinct clinical phenotypes providing insight into clinical course, preferred treatment and prognosis [4–7].

Recent developments in the evaluation of myositis specific autoantibodies (MSAs) have led to the categorization of JIIMs as a heterogeneous group of illnesses characterized by varying clinical phenotypes, organ involvement, and prognosis (Table 1). Anti-p155/140 autoantibodies, most commonly seen in white patients, are associated with significant cutaneous involvement, generalized lipodystrophy and a chronic disease course in approximately 65% of patients [4]. Patients with anti-MJ autoantibodies have significantly increased risk of calcinosis compared to those with other autoantibodies [10]. Anti-synthetase autoantibodies are associated with mechanic’s hands, arthralgias and interstitial lung disease (ILD), and the highest mortality rate among the MSA phenotypes (16.7%). Conversely, anti-Mi2 autoantibodies are often associated with pathognomonic JDM rashes and a better prognosis relative to other MSA phenotypes [4]. Anti-MDA5 antibodies, a more recently described MSA, are associated with skin ulceration, amyopathic course and rapidly progressive ILD [6]. Myositis associated autoantibodies (MAAs), including anti-polymyositis scleroderma (PmScl), anti-U1 ribonucleoprotein (U1RNP), and anti-Ro, have also been described in association with JIIMs as well as with overlapping connective tissue diseases [7].

**Table 1:** Clinical Features Associated with Myositis Specific Autoantibodies

Myositis Specific Autoantibody	Anti-p155/140 [8]	Anti-MJ	Anti-synthetase *(including anti-Jo-1)*	Anti-Mi-2	Anti-MDA-5 [9]
Autoantigen Target	TIF-1	NXP-2	Histidyl-tRNA synthetase	Mi-2	CADM140
Frequency	18–30%	15–20%	2–4%	2–10%	6% (UK) – 38% (Japan)
JIIM subgroup (predominant)	JDM	JDM > JPM	JDM > Overlap CTD > JPM	JDM	JDM
Median age at onset (years)	7 (4–10)	6 (4–8)	14 (10–14)	11 (6–12)	6 (4–10)
Race/ethnicity	White	White	White>Black	Hispanic	Variable, case reports in Japan
Skin	Classic rash, photosensitive rash, cutaneous ulceration	Classic rash	Mechanics hands, Raynauds	Classic rash	Palmar papules, cutaneous ulceration
Weakness	Moderate	Severe	Mild/moderate	Mild	May be amyopathic
Muscle enzyme elevation	Low	Moderate	Moderate	High	Mild
Additional musculoskeletal features	Muscle atrophy	Muscle cramps, joint contractures	Arthritis/ arthralgias	Less Common	Arthritis
Major organ involvement	Less common	Dysphagia, GI ulceration	Interstitial lung disease	Less common	Interstitial lung disease
Complications	Calcinosis (30%), lipodystrophy	Highest rate of calcinosis (47%)	Less calcinosis (10%), lipodystrophy (33%)	Calcinosis (18%)	Calcinosis (27%)
Prognosis	Chronic course, low mortality	Chronic course, 1/3 monocyclic, low mortality	Highest risk of mortality	Best prognosis	Poor prognosis in Japan cohort
References	4,5,7,11	4,5,7	4,5,7	4,7	6,7, 9,10,12

*Abbreviations*: *Anti-TIFI, anti-p155/140*, Anti-transcription intermediary factor 1 gamma, *Anti-NXP2, anti-mj*, Anti-nuclear matrix protein 2, *anti- MDA5* anti-melanoma differentiation associated protein 5

Despite the growing literature surrounding MSAs in patients with JIIM, the majority of studies include predominantly white populations with limited data on the extent to which race and ethnicity impact disease manifestations, treatment response and long-term clinical outcomes. We report a retrospective case series of pediatric patients with JIIM to determine the prevalence of MSAs and MAAs, and associated clinical phenotype and disease course in a racially diverse pediatric population.

## Methods

A retrospective chart review was completed at the Children’s Hospital at Montefiore, collecting clinical and laboratory data via manual chart review of the electronic medical record from the time of diagnosis to the time of data completion in January 2020. Patients age 2–21 years old with a prior diagnosis of JDM, JPM or overlap myositis, who had been tested for MSA/MAA were eligible for study inclusion. One patient with JDM did not have MSA/MAA testing and was thus excluded. Race and ethnicity were self-identified and reported as white, Black or Hispanic. No patients identified as more than one category. Study approval was obtained via the Einstein Institutional Review Board (IRB), and all data were collected and maintained in a confidential fashion. A waiver of informed consent and HIPAA authorization were approved by the IRB. Patient demographics, clinical manifestations, organ involvement, treatment and disease course were recorded. Muscle strength was assessed using the Childhood Myositis Assessment Scale (CMAS) and Manual Muscle Testing (MMT). Descriptive statistics were performed to summarize each variable. Given the small sample size, non-parametric testing was performed using Fischer’s exact test, Wilcoxon rank sum test and Kruskal-Wallis test. We additionally describe two complex cases of children of Black or Hispanic descent with anti-MDA5 antibody positivity.

## Results

A total of 31 patients were included in the analysis. Overall median age at diagnosis was 7 years (range 2–16), and the majority were female sex (80.7%). Race and ethnicity were self-reported as Hispanic (48.4%), white (25.8%), and Black (25.8%). The most prevalent MSAs were anti-MDA5 (25.8%), anti-p155/140 (22.6%) and anti-MJ (19.4%), followed by anti-Mi2 (3.2%), anti-synthetase (3.2%) and several MAA. Six patients were weakly positive for a second MSA/MAA, however only the predominant antibody was included in our current study. Patient demographics and disease features, grouped by race and ethnicity, are shown in Table 2. Presence of autoantibodies (*p* = 0.04) and pulmonary disease (*p* = 0.03) were significantly higher in patients of Black or Hispanic descent. Male gender, cutaneous ulceration, hospitalizations and death were only present in patients of Black or Hispanic descent. Interestingly, there was a relatively high proportion of patients with anti-MDA5 antibodies in our cohort (*n* = 8) and all were of Black or Hispanic descent. Patients with anti-MDA5 antibodies were more likely to be of male sex (p = 0.04) and to have cutaneous ulceration (*p* = 0.02). Of the 11 patients with pulmonary involvement, 5 had anti-MDA5 antibodies (*p* = 0.24). Two patients demonstrated unique clinical courses of JDM associated with Black or Hispanic descent and anti-MDA5 antibody presence.

**Table 2:** Patient characteristics in 31 children with idiopathic inflammatory myositis^a^

	White (n = 8)	Black/Hispanic (*n* = 23)	*p*-value
Age at diagnosis (years)	8.5 [3,11]	4 [3,9]	0.31
Female sex	8 (100%)	17 (73.9%)	0.30
Diagnosis			
JDM	8 (100%)	20 (87%)	0.55
Overlap/JPM	0	3 (13%)	
Disease duration (months)	48 [18,102]	27 [15,73]	0.22
Weakness			
None/mild	6 (71.4%)	14 (70%)	> 0.99
Moderate/severe	2 (28.6%)	5 (30%)	
Skin rash			
None	0	5 (25%)	0.48
Typical	6 (85.7%)	11 (55%)	
Atypical	1 (14.3%)	4 (20%)	
MSA/MAA			
Antibody negative	2 (25%)	0	**0.04**
Anti-MDA5	0	8 (34.8%)	
Anti-p155/140	3 (37.5%)	4 (17.4%)	
Anti-MJ	3 (37.5%)	3 (13%)	
Anti-Mi2	0	1 (4.4%)	
Anti-synthetase (PL-12)	0	1 (4.4%)	
Anti-Ro	0	4 (17.4%)	
Anti-RNP	0	1 (4.4%)	
Anti-PM-Scl	0	1 (4.4%)	
Disease features			
Ulceration	0	7 (33.3%)	0.14
Calcinosis	2 (28.6%)	7 (33.3%)	> 0.99
Lipodystrophy	0	3 (14.3%)	0.55
Gastrointestinal	1 (14.3%)	8 (38.1%)	0.37
Cardiac	0	3 (13.6%)	> 0.99
Pulmonary	0	11 (50%)	**0.03**
Disease Course			
Hospitalizations^b^	0	7 (38.1%)	0.08
Death	0	1 (4%)	> 0.99

*Abbreviations*: *JDM* Juvenile Dermatomyositis, *JPM* Juvenile Polymyositis, *MSA* Myositis-specific autoantibody, *MAA* Myositis-associated autoantibody

^a^Variables are expressed as median (interquartile range) and frequency (percentages)

^b^Represents the number of patients requiring hospitalization during their disease course

## Case one

A Black female who initially presented at 23 months of age with atypical rash (purpuric hyperpigmentation diffusely on body and face), fever and decreased ambulation. Labs were notable for leukopenia, elevated lactate dehydrogenase (LDH) and Von Willebrand Factor (VWF) antigen and positive anti-nuclear antibody (ANA). Liver findings included elevated transaminases, cholestasis, and ultrasound findings of mild echogenicity. A liver biopsy revealed inflammation but was not consistent with autoimmune hepatitis or sarcoidosis. She subsequently developed subcutaneous nodules on upper arms and legs, which excisional biopsy revealed as fat necrosis and micro-calcifications. An MRI of the pelvic girdle revealed bilateral patchy areas of muscle edema and mild subcutaneous edema consistent with JDM. EMG was normal, however limited, as it was performed under sedation. This patient had a rapidly progressive disease course and within 1.5 months of symptom onset she was admitted to the intensive care unit for respiratory distress. Treatment included methylprednisolone, IVIG and rituximab. She developed progressive respiratory failure requiring intubation and mechanical ventilation. Bronchial alveolar lavage fluid resulted positive for pneumocystis jiroveci pneumonia (PJP) and therapy was initiated. Her course was complicated by pneumomediastinum. On hospital day #16 she underwent pulseless electrical activity cardiac arrest secondary to hypoxic respiratory failure, unresponsive to CPR, and died at 24 months of age. Left quadriceps muscle biopsy, which resulted post-mortem, was notable for abnormal immunohistochemical staining for MHC class I antigens and MAC C5b-9, suggestive of myopathic process, as well as mild type 2 myofiber atrophy. MSA panel resulted post-mortem and was notable for positive anti-MDA5 antibody. Lung pathology post-mortem showed evidence of underlying ILD. Of note, her respiratory symptoms preceded initiation of immunosuppressant therapies suggesting that PJP might have been acquired prior to treatment for JDM. Comprehensive work-up for underlying primary immunodeficiency and genetic etiologies were performed during hospitalization and were unrevealing.

## Case two

A Hispanic female who initially presented at 3 years of age with an excoriated, atypical vesicular rash, coalescing into plaques on upper arms, thighs, calves and lower buttocks. Infectious work-up was negative. Skin biopsy revealed superficial and deep perivascular dermatitis with neutrophils. She additionally had decreased ambulation, abnormal liver echogenicity, and elevated transaminases. A liver biopsy was notable for micro & macrovesicular steatosis without fibrosis, and muscle biopsy was normal. MRI was significant for subcutaneous and fascial edema, concerning for JDM. Patient also suffered from cardiac involvement including ectopic atrial tachycardia (EAT) and decreased left ventricular systolic function. She was found to be anti-MDA-5 positive and her treatment regimen included corticosteroids, hydroxychloroquine, cyclophosphamide induction and maintenance therapy with mycophenolate mofetil. She underwent ablation of her EAT and has no pulmonary involvement to date. At 4 years from the time of diagnosis, significant calcinosis cutis has been her only persistent JDM manifestation.

## Discussion

This study demonstrates a wide variety of MSA/MAA in a racially diverse group of patients with JIIM and further delineates clinical phenotypes and disease complications in these groups. We provided an in-depth case description of two young females, one of Black descent and one of Hispanic descent, with anti-MDA5 autoantibodies presenting with atypical rash and prominent liver involvement. One patient had rapidly progressive pulmonary involvement with ultimately fatal disease, the other patient suffered from significant cardiac involvement. Among the JDM patients of Black or Hispanic descent in our cohort, one third (34.8%) were found to have evidence of anti-MDA5 antibodies, which is higher than previously reported in other patient populations described in the literature. In a study performed by Tansley et al., composed primarily of white patients (76%), anti-MDA5 antibodies were identified in only 7.4% of JDM patients and were associated with distinct clinical phenotypes, including skin & oral ulceration, arthritis, and milder muscle disease (compared with patients without MDA-5 antibodies). In this study, 19% of patients with anti-MDA5 antibodies had evidence of ILD and none with rapidly progressive disease [6]. Hoshino and colleagues examined 135 adult Japanese patients with connective tissue disorders and found that 26% of patients with dermatomyositis (*n* = 82) had anti-MDA5 positivity [11]. These findings, in conjunction with this current study, suggest a possible increased incidence of anti-MDA5 presence in patients of non-white descent.

Research in the adult population of dermatomyositis has suggested that anti-MDA5 antibody presence is associated with increased risk of ILD, however, these studies are also in primarily white and Asian populations [11, 12]. In our study, 5 out of the 8 children with anti-MDA5 antibodies were found to have evidence of ILD. In the cases highlighted in our current study, two patients of non-white descent had an atypical disease presentation, delay of diagnosis and severe disease course associated with anti-MDA5 antibody presence. Interestingly, these patients both had notable liver involvement, which has not yet been described in association with anti-MDA5 antibody presence. This suggests that autoantibody testing early in the disease course could not only help with diagnosis but may also be a valuable tool in providing anticipatory management for these patients; in particular for the prompt recognition and treatment of end-organ involvement. Understanding racial differences in regards to MSA presence, organ involvement and disease prognosis is important in order to appropriately diagnosis, screen and treat various JIIMs.

There are important limitations to our study. First, this was a retrospective study on a small sample size, with two anecdotal cases presented and thus true associations between race, MSA/MAA and disease features JIIM cannot be conclusively determined. Another limitation is that all patients self-identified as white, Black or Hispanic, with only one category selected. It is unknown, if given the option, patients may have selected more than one race/ethnicity and whether this could have impacted the results. Additionally, we did not analyze or account for socioeconomic status, which may play a role in disease presentation and course separate from race and ethnicity. Finally, few patients had evidence of an additional, weakly positive MSA, however these were not considered for the purpose of the analysis. The role of these additional, weakly positive, MSA is unknown.

## Conclusions

This study is novel in that it details pediatric patients with JIIM and MSA/MAA presence in a population that is primarily Black and Hispanic. Despite the rarity of JIIM, we found a relatively high proportion of children with anti-MDA5 antibodies and have described potentially worse clinical outcomes in children of Black or Hispanic descent, especially in those with positive anti-MDA5 antibodies. Further investigation is warranted to examine these findings in a larger sample of patients.

## Data Availability

The datasets generated and analyzed during the current study are not publicly available due to HIPAA but are available from the corresponding author on reasonable request.

## Abbreviations

Anti-TIFI, anti-p155/140: Anti-transcription intermediary factor 1 gamma
Anti-NXP2, anti-mj: Anti-nuclear matrix protein 2
anti- MDA5: Anti-melanoma differentiation associated protein 5
JDM: Juvenile Dermatomyositis
JIIM: Juvenile Idiopathic Inflammatory Myopathy
JPM: Juvenile Polymyositis
MAA: Myositis-associated autoantibody
MSA: Myositis-specific autoantibody
